# Evaluation of prevention of mother-to-child transmission national health information system for HIV/AIDS, in southern region of Mozambique, April to November 2016

**DOI:** 10.11604/pamj.2021.38.26.24255

**Published:** 2021-01-12

**Authors:** Auria Ribeiro Banze, Benilde Pedro Homo, Tufária Nazimo Mussá, Cynthia Semá Baltazar, Makini Aida Boothe

**Affiliations:** 1 Mozambique Field Epidemiology and Laboratory Training Program, National Institute of Health, Maputo, Mozambique,; 2 National Institute of Health, Maputo, Mozambique,; 3 Program for the Prevention of Mother to Child Transmission, National Directorate of Public Health, Ministry of Health, Maputo, Mozambique,; 4 Department of Microbiology, Faculty of Medicine, Eduardo Mondlane University, Maputo, Mozambique,; 5 Global Health Sciences, University of California, San Francisco, Maputo, Mozambique

**Keywords:** Mother-to-child transmission, surveillance system, HIV Infection, Mozambique

## Abstract

**Introduction:**

Mozambique has a generalized HIV epidemic, among pregnant women, HIV prevalence is estimated at 15.8% with a vertical transmission rate of 14%, more than double global targets. We evaluate electronic national health information system (SIS-MA) performance to verify if the data flow procedures met its objectives and evaluated the prevention of mother-to-child transmission (PMTCT) surveillance system to access its attributes and usefulness.

**Methods:**

we conducted a descriptive, cross-sectional evaluation of the PMTCT surveillance system in eight facilities in Gaza and Inhambane provinces using the centers for disease control and prevention guidelines (2001). For data quality, we cross-referenced patient registries from health facilities against the SIS-MA. We also interviewed 34 health technicians, using a Likert scale, to assess the following attributes of the PMTCT surveillance system: simplicity, stability, flexibility, acceptability, timeliness and data quality, usefulness of the system and knowledge of PMTCT.

**Results:**

regarding the simplicity measure, we verified that the registry books contain more than 30 variables. The system was 83% flexible in maintaining functionality with the introduction of new health facilities in the system. The completeness of the data was 50% and concordance of data from the register book and monthly reports was 89%.

**Conclusion:**

the PMTCT SIS-MA is useful in supporting the collection, analysis, interpretation and continuous and systematic dissemination of health data that are used to define and monitor public health policies in Mozambique. However, continued efforts are needed to improve data quality to ensure that the SIS-MA can adequately monitor the PMTCT program and contribute to reduced vertical transmission.

## Introduction

Despite a concentrated response to HIV/AIDS over the last decade, the epidemic remains a public health threat with significant rates of morbidity and mortality at a global level [1] and especially in the sub-Saharan African region where HIV/AIDS disproportionately affects women of reproductive age [2]. Mozambique has one of the highest HIV prevalence estimates in the region, with 13.2% in the general population aged 15-49; among the same age group, the prevalence is higher among women (15.4%) than men (10.1%) [3]. There is an estimated prevalence of 15.8% among pregnant woman and a vertical transmission rate of 14%, which is more than double global targets [4]. Prevention of mother-to-child transmission (PMTCT) has been one of the key interventions of the HIV/AIDS response. Option B+ services, universal antiretroviral treatment for HIV-infected pregnant and breastfeeding women, were introduced in Mozambique in 2013 as a component of AIDS national strategic plan (NSP) to enhance services targeting pregnant women with focused attention on the reduction of vertical transmission [5].

In an effort to reach global targets and ensure the existence of robust data collection systems, the Mozambican ministry of health (MOH) introduced longitudinal register books in April 2016 to monitor option B+ implementation and improve the quality of the PMTCT reporting system [6]. During the same period, the electronic information system for monitoring and evaluation (SIS-MA), was developed, based on a DHIS2 platform, to respond to the growing demand for information and to improve the information data flow from the health facility (HF) to the national level. It was envisioned that this electronic system would support the use of routine PMTCT data for surveillance purposes by providing the necessary data to track trends and monitor the course of the epidemic, measuring the effectiveness of prevention and treatment interventions and supporting the planning of more effective HIV response efforts.

**Figure 1:**
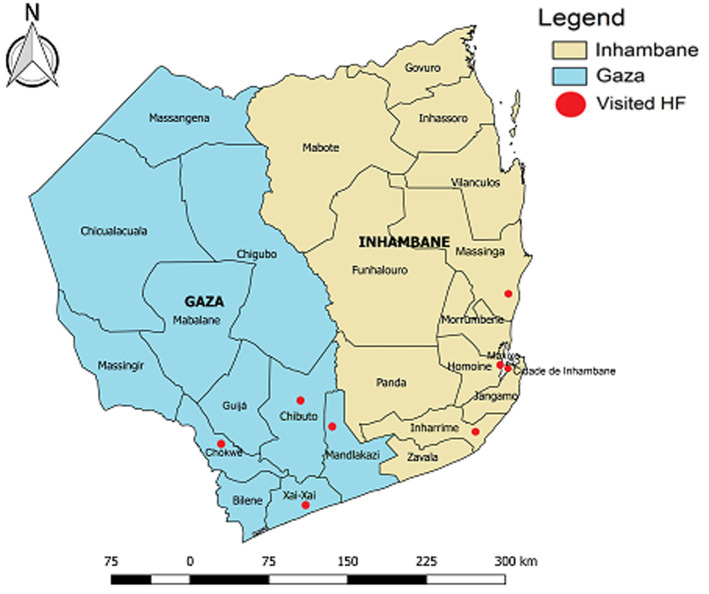
health facilities at which the PMTCT national health information system for HIV/AIDS was evaluated Gaza and Inhambane provinces, Mozambique, 2016

PMTCT data is generated and recorded for each patient by nurses at each health facility on a daily basis. At the monthly reporting period (20^th^ of every month), the nurse responsible for PMTCT services in each health facility compiles the data and sends it to the district statistical team by the 23^rd^ day of each month. The district statistical team then enters the data into SIS-MA and a summary is sent to the province by the 28^th^ of each month. At the provincial statistical center, the data is analyzed and input into the SIS-MA. A summary of the data is provided to the district. The department of health Information analyses the data and sends back information to the provincial statistical nuclei until day 15 of the following month. In the last steps, the national HIV program at the central level analyses the data and sends back information to the health Information department and provincial supervisors (Figure 1).

Evaluating the use of SIS-MA in the context of the PMTCT information system leads to a better understanding of one essential component of Mozambique´s HIV surveillance system [7]. In this context, the purpose of this analysis was to evaluate the SIS-MA to verify if the data flow procedures meet its objectives in reducing vertical transmission. We also provide recommendations for improvements at different levels of the system.

## Methods

**Study design:** we carried out a descriptive, cross-sectional evaluation of the PMTCT surveillance system between April and November 2016. The evaluation of the system was conducted per centers for disease control and prevention (CDC) guidelines (2001), which outline key attributes of the system including: simplicity, flexibility, data quality, acceptability, sensitivity, predictive positive value, representativeness, timeliness and stability. For this evaluation, we focused on the attributes of simplicity, flexibility, data quality, acceptability, representativeness, timeliness and stability using observation, data review (PMTCT register books, monthly summary reports and the SIS-MA database) and interviews. It was not possible to evaluate the sensitivity and the predictive positive value due to the absence of variables to evaluate these attributes in the system.

**Study site:** the study was conducted in Gaza and Inhambane provinces, located in the southern region of Mozambique (Figure 1). We selected these provinces because they are located in the southern region, which has the highest prevalence of HIV infection among pregnant women (23.6%) in Mozambique [8].

**Study population, sample size consideration and sampling technique:** using a semi-structured questionnaire, we interviewed 28 maternal and child health (MCH) nurses responsible for PMTCT in the eight health facilities and eight district-level health statisticians responsible for entering data in SIS-MA. The questionnaire assessed their level of knowledge about the system and evaluated the following attributes: simplicity, flexibility, acceptability, representativeness, timeliness, stability and usefulness. We selected at convenience sample of four health facilities (HF) from Gaza and Inhambane provinces. These facilities reported the highest numbers of MCH patients in 2016 and also offered antiretroviral treatment (ART) in the antenatal consultation (ANC) and HIV exposed infants/at-risk children services [7]. For the review of the option B+ registry books and monthly SIS-MA summary reports, one month was randomly selected for each health facility from April to December 2016.

**Data analysis:** the interviews were first transcribed. The interviews were then coded and then we analyzed knowledge and system attributes, as shown in the panel below. We used the Likert scale, a psychometric response scale used in questionnaires and opinion surveys in which the interviewees specified their level of agreement with a statement, based on a scale [9]. The Likert scale is a verified tool used as a basis to measure parameters similar to ones in this evaluation [10-12]. Data was analyzed using SPSS 16.0, descriptive analysis presented in frequency tables and for the evaluation of attributes, parameters described in a table using Microsoft Excel were used.

### Variable measures

**Simplicity:** structure and ease of operation of the health information system [13]. The following elements were evaluated for simplicity: number of key variables collected from the register books; number of documents to be filled out; data flow (health facility>district>province>national); number of trained personnel. Score: 1 to 8 classification: simple=2 points; complex=1 point. Final score: simple: ≥6 points; complex <6 points [11].

**Flexibility:** the system´s ability to adapt to changes in information needs. We observed the ability of the system to remain functional during the introduction of new health facilities and the change of trained personnel. Final score: yes: flexible or no: not flexible.

**Acceptability:** the willingness of individuals and institutions to use the surveillance system. We evaluated the interest of the professionals participating in the system (based on the data notification rate) and the opinion of the technicians about the registration and data entry in the system. Final score: low: <70%; moderate: 70 to 89%; high: ≥90% [10].

**Representativeness:** the ability of the system to accurately describe the occurrence of a health-related event over time and its distribution in the population by place and person. Person: age, sex, clinical status; time: date of 1^st^ consultation; place: province, district, health facility, residence. Final score: high: ≥85%; low: <85%.

**Timeliness:** the time required to accomplish the various steps in a surveillance system. We analyzed the amount of time it took to fill out the register book (timely - up to 10min; >10min - not timely); the time interval between the aggregation of the data in the monthly report and the reporting to the district statistics team (≤1 week = timely; ≥1 week = not timely); the time necessary to enter the data in the system (until 24 hours = timely; >24 hours = not timely). Final score: timely: ≥50% of stages satisfactory; mot timely: ≤50% of stages.

**Stability:** the ability of the system to be operational when needed. We evaluate the interruption of the system from the beginning of the functionality; availability of the data in the system after the introduction of the same in the database; capacity of the SIS-MA to be operational even if it is offline. Final score: high =yes; low =no.

**Usefulness:** an ability of the system to contribute to the prevention and control of adverse health events, including a better understanding of the public health implications of such events: we evaluated the ability of SIS-MA to support the collection, analysis, interpretation and continuous and systematic dissemination of health data. Final score: yes=useful; no= not useful [11].

**Data quality:** the integrity and validity of the data recorded in the system. We evaluate the number of variables/fields filled out with in completing the data. Data concordance we compared the data from the book, monthly summary and system database. Score: low <70%; moderate - 70 to 89%; high ≥90%.

**Knowledge of the health professionals:** the knowledge of procedures for various PMTCT services: counselling; analysis of CD4, hemogram and biochemistry; the type of ART provided, prophylaxis with cotrimoxazole and isoniazid; early infant diagnosis; exclusive breastfeeding; childbirth; invitation of the partner for testing. Final score: low 0 - 2, acceptable 3 - 4 and good 5 - 7.

**Ethical considerations:** ethical approval was obtained from the institutional committee of bioethics in health of the Faculty of Medicine and Maputo Central Hospital (CIBS FM & HCM). We obtained informed written consent from the interviewees. Participation was voluntary and anonymity was guaranteed by not indicating the interviewees´ name on the questionnaire. We coded the identification (name) of the participants so that there is a link between the code and the information.

## Results

**Characteristics of the study participants:** of the 34 health workers interviewed, 85% were female, with a median of 9 years of service. The majority (82%) of the respondents were MCH nurses, while 6/34 (18%) were district health statisticians (Table 1). Of the 28 MCH nurses, 18/28 (64%) provided ANC services and 10/28 (36%) worked with HIV-exposed infants/at-risk children.

**Table 1:** demographic characteristics of health workers in southern Mozambique, 2016

Characteristics	Frequency (%) (n=34)
**Gender**	
Male	5 (15)
Female	29 (85)
**Qualification**	
MCH nurse - ANC	18 (64)
MCH nurse - HIV exposed infants/at-risk children	10 (36)
Health statistics technicians	6 (18)

**Simplicity:** we found that the ANC registry books contain two pages for each patient, where 53 variables are collected across 15 categories: date of visit; demographic variables; number of medical visits; pregnancy stage; partner; nutritional assessment; physical or laboratory examination; screening and treatment of sexually transmitted infections; HIV status; malaria; prevention of postpartum hemorrhage; anti-tetanus vaccine; tuberculosis screening; notes section for additional observations; and legible name of the healthcare professional. The children's reference book also contains two pages for each patient with 46 variables across 11 categories: child´s demographic information; mother's demographic information; risk condition; tuberculosis screening; child´s nutritional assessment; child exposed to HIV; rapid HIV test result for the unexposed child; discharge of the child from outpatient consultation; patient ID; notes section for additional observations; legible name of the healthcare professional.

**Figure 2:**
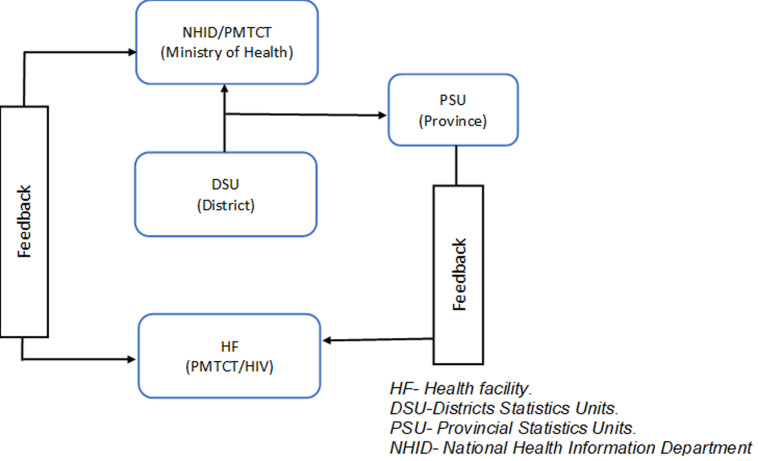
description of PMTCT information flow, Mozambique, 2016

Eighty-two percent (23/28) of the nurses stated that there were additional data collection tools to fill out for each patient including: registry books for counseling and testing, sexually transmitted infection, intermittent presumptive treatment, viral load, CD4 and gender-based violence (GBV). Separate registry books were also required for nutritional rehabilitation program, polymerase chain reaction (PCR) HIV-exposed infants/at-risk children and isoniazid prescriptions. Sending the data after collecting it in the registry books was considered complex due to the large volume of data collected daily (Table 1). The majority of trained personnel reported that the system is simple to use in the organizational chart demonstrated the PMTCT flow of information from the health facility to the MOH (Figure 2). Based on the scoring method used in the Likert scale, the nurses and statisticians classified the system as simple (with 7 points for the evaluated items).

**Flexibility:** of the statisticians interviewed, 83% (5/6) reported that the electronic reporting system was functional, given its ability to remain online even when new health facilities were entered into the database. Eighty-two percent (28/34) of the interviewees stated that it was possible to make staff changes as long as there were persons trained in data collection and entry (Table 2). As a result, system was classified as flexible.

**Table 2:** results of the simplicity, flexibility and acceptability questions of the PMTCT in southern Mozambique, 2016

Variable	Response options	Frequency (%)
**Simplicity**		
Number of key variables for data collection in the register books	More than one logbook	28/28 (100)
Do you have more than one registration book to complete	Yes	23/28 (82)
Registration book data submission level	Three levels	28/28 (100)
Number of trained personnel	All	28/28 (100)
Time used for system maintenance	Less than a week	6/6 (100)
**Flexibility**		
SIS-MA ability to remain functional with the introduction of new HF	Yes	5/6 (83)
The possibility and the flexibility of changing the person responsible for data entry and reports	Yes	28/34 (82)
**Acceptability**		
What do you think about the registry book	It's very good, facilitates the follow-up, appropriate monitoring of pregnant woman, breastfeeding and the child; excellent, improves quality services, in data collection, quicker to register patients	12/28 (43)
What do you think about the SIS-MA database	It's good, good data aggregator, facilitates data analysis and interpretation, helps to manage information, but needs to be improved in relation to the maintenance of the system to avoid oscillation and frequent interruptions and introduction of more data validation rules in the system	4/6 (67)

**Acceptability:** we verified that data on all patients admitted to the health facility and registered in the registration books, was sent to the district health statistics units (100%). Forty-three percent (12/28) of the nurses positively favored the registry book because it facilitates monitoring pregnant and breastfeeding women and their children. Sixty-seven percent (4/6) of district-level health statisticians stated that the SIS-MA database was a good aggregator of data and facilitated data analysis and interpretation. They further expressed that it helps to manage information quickly but to system maintenance needs to be improved to avoid frequent interruptions due to unstable internet connection. It would also benefit from additional data validation rules and logic checks to avoid duplications or implausible entries (Table 2). During interviews, we found that all nurses have a lot of new patients in the consultation. Based on the evaluated parameters, SIS-MA has a moderate acceptability with a score of 70%.

**Representativeness:** mandatory epidemiological variables were collected for all patients initiating antiretroviral therapy in the ANC and HIV-exposed infants/at-risk children services who attended the consultation. Thus, the system demonstrated high representativeness of the PMTCT patient population.

**Timeliness:** it took the nurses, on average, seven minutes to fill out the registry book per patient. At the close of the programmatic month, all nurses aggregated the data in the monthly report and sent it to the district-level statistical units within a week. The statisticians then took an average of 8 minutes to enter the monthly summary in the SIS-MA, where the data was automatically aggregated by district, provincial and national level. The automated aggregate processes demonstrate that the system is timely.

**Stability:** all six statisticians stated that the system lost internet connection at least once a week. While offline, data is inserted and later uploaded to the server when connectivity returns. If the system was interrupted more than 2 times in the same month, it could be interrupted for a week. Given these limitations, the system had low stability.

**Usefulness of the system:** the system is capable of detecting cases of HIV in the provinces, the number of new cases of HIV in pregnant women and in newborn children and regions most affected by HIV in Mozambique. The data is used at several levels through the health system - site, district, provincial and national levels - to support program monitoring. As such, we classified SIS-MA as 100% useful.

**Data quality:** in the registry books, we found 265/401 (66%) blank fields and 326/401 (81%) indicators were concordant with the monthly reports. Overall, the data concordance was of 89 percent between data reported in the register books and monthly reports. As such, the data quality of this system is considered moderate, with a score of 79%.

**Knowledge of the nurses about PMTCT:** according to the interviews, 26/28 (93%) of the nurses correctly answered questions related to the type of ART provided, prophylaxis with cotrimoxazole and isoniazid; 19/28 (68%) about counseling and testing; 20/28 (71%) about CD4 analysis, hemogram and biochemistry; 22/28 (79%) about early infant diagnosis; 5/28 (18%) about exclusive breastfeeding; 6/28 (21%) about childbirth and 8/28 (29%) about inviting the patient´s partner for testing (Table 3). According to the Likert scale, 12/28 (43%) of the nurses showed good knowledge of PMTCT.

**Table 3:** PMTCT knowledge of nurses, southern Mozambique, 2016

Variable	Frequency (%)
Knowledge of PMTCT services	
Counseling and testing	19/28 (68)
Analysis of CD4, hemogram, and biochemistry	20/28 (71)
The supply of ART, prophylaxis with cotrimoxazole and isoniazid	26/28 (93)
Early Infant Diagnosis	22/28 (79)
Exclusive breastfeeding	5/28 (18)
Childbirth	6/28 (21)
Invitation of the partner for testing	8/28 (29)

## Discussion

This evaluation demonstrated the important role of SIS-MA as a tool with the potential to improve the PMTCT reporting system. Although we concluded that the system was simple, there are numerous variables distributed across various registry books that need to be filled out during each clinical consultation. A study in South Africa pointed out that the simplicity of paper-based registry tools presents limitations as patient volumes increase [14]. This challenge is common particularly in ANC, which 50 or more new patients initiate treatment per month in a single HF [14].

The system was considered flexible since it easy to enter new health facilities once the system became functional, facilitating a rapid return of information for timely decision-making. New systems can be modified rapidly, without the loss of information, which is one of the most fundamental qualities of any health information system [12].

Nurses occasionally did not complete the registry book in an acceptable manner due to the high demand of new patients and the lack of technical support. There was a shortage of staff to support all the activities of the consultation, from counseling the patient to the registry of data. According to the ministry of health, the average maternal child health nurse ratio is one per 4000 inhabitants; however, the World Health Organization (WHO) recommends one nurse per 1000 inhabitants [15]. Poor job allocation, high turnover rates, low retention in the national health system and the low profitability of health facility staff all hamper the provision of quality services. Additionally, the lack of mentoring, continuous training and supervision compete with the nurses´ ability to attend to patients and implement all of the required services [16].

Regarding data quality, we found many blank fields in the registry books, showing a low completeness of the system. Our results are similar to a study conducted in South Africa, where they found that the data elements were only half complete [17]. Another South African study, which had a relatively high completeness (91%), revealed considerable data quality concerns for PMTCT information and an average accuracy of data between the registration and monthly report of 51% [18]. In contrast, we found high data concordance after comparing the registry book and monthly summary. These findings are in accordance with a study in Mozambique that the concordance of the clinical record data with the monthly reports was good [19].

In this evaluation we found the data flow from the registry book to the database of regular data quality. The low-level of data agreement is one of the main PMTCT data quality concerns and despite the increase in PMTCT coverage over the period, the variation in data raises concerns about the reliability of the information. The system is timely and useful and has demonstrated of its ability to collect, analyze, interpret and continuously disseminate ANC and HIV exposed infants/at-risk children data in relation to PMTCT, reflecting the magnitude of HIV morbidity and mortality in women and children in the country. However, the system had a low stability due to frequent internet outages. System interruptions are frequent, mainly at the end of each month, making it difficult to enter monthly data into the system.

This evaluation shows that the PMTCT information system should be reviewed from the health facilities´ patient registries to the SIS-MA programmatic reporting to ensure better quality data collection and improved monitoring of HIV cases in pregnant women, infants and children. This will improve decision-making and public health action to reduce HIV/AIDS morbidity and mortality and improve the health of the general population. Despite the important elements outlined above about the strengths of the current PMTCT surveillance system, our study had a few limitations. We were not able to evaluate the sensitivity, positive predictive value and costs of the health information monitoring and evaluation system, which was started in maternal and child health consultations, because there were no data available. During the interviews, there was potential information bias of some nurses due to the workflow that impacts their memory and lack of availability of some statistical technicians. SIS-MA was also difficult to assess in some districts due to system interruption. We attempted to address this challenge by conducting the interviews with the nurses after they had attended to all the patients so that they were able to answer the questions without interruption.

## Conclusion

The PMTCT SIS-MA is useful; it supports the collection, analysis, interpretation and continuous and systematic dissemination of health data that are used to define and monitor public health policies. The demand of new patients in the consultation room and the lack of technical support can influence the moderate acceptability of the system among nurses and the quality of data because the nurses have a large workload. However, continued efforts are needed to improve data quality to ensure that the SIS-MA can adequately monitor the PMTCT program and contribute to reduced vertical transmission.

### What is known about this topic

- The SIS-MA in HIV PMTCT is responsible for supporting the collection, analysis interpretation, continuous and systematic of ANC and HIV exposed infants/at-risk children service data in relation to PMTCT;
- The SIS-MA in HIV PMTCT is important for monitoring HIV cases in pregnant women, nursing mothers and children for public health action with the aim of reducing the morbidity and mortality of HIV / AIDS and improving the health of the general population.

### What this study adds

- The study shows that the SIS-MA in HIV PMTCT can be considered as a hospital-based surveillance system as it manages to demonstrate its magnitude of HIV morbidity and mortality in women and children in the country;
- This study demonstrates the importance of periodically conducting SIS-MA assessments in PMTCT, especially after its implementation, in order to contribute to the improvement of the system and consequently, to improve the country's public health.

## References

[1] UNAIDS (2019). Global AIDS update 2019 - communities at the centre - defending rights. breaking barriers reaching people with HIV services.

[2] Moçambique Ministério da Saúde (2013). Plano estratégico do sector da saúde. PESS 2014-2019.

[3] Mocambique Instituto Nacional de Saude Instituto Nacional de Estatistica (2015). Inquérito de indicadores de imunização. malária e HIV/SIDA em Moçambique (IMASIDA).

[4] MISAU Instituto Nacional de Estatística (INE) (2010). Inquérito nacional de prevalência riscos comportamentais e informação sobre o HIV e SIDA em Moçambique 2009. Moçambique Ministério da Saúde.

[5] MISAU (2014). Relatório anual das actividades relacionadas ao HIV/SIDA.

[6] UNAIDS WHO (2013). Evaluating a national surveillance system. World Health Organization.

[7] MISAU (2017). Relatório anual 2016: relatório anual das actividades relacionadas ao HIV/SIDA 2016.

[8] Instituto Nacional de Saúde Instituto Nacional de Estatística e Grupo Técnico Multisectorial de Apoio à Luta Contra ao HIV/SIDA em Moçambique (GTM Ministerio Da Saude) (2013). Ronda de vigilância epidemiológica do HIV e sífilis em Moçambique 2011.

[9] Joshi A, Kale S, Chandel S, Pal D (2015). Likert scale: explored and explained. Current Journal of Applied Science and Technology.

[10] Meirelles MQB, Lopes AKB, Lima KC (2016). Vigilância epidemiológica de HIV/Aids em gestantes: uma avaliação acerca da qualidade da informação disponível. Rev Panam Salud Publica.

[11] Mota DM, Freitas DRC, Araújo WN (2012). Avaliação do Sistema de Vigilância Sanitária do Sangue em âmbito federal Brasil 2007. Cien Saude Colet.

[12] Barbosa JR (2011). Avaliação do sistema de vigilância epidemiológica da dengue no Brasil 2005-2009. Goiania.

[13] Centers for Disease Control and Prevention (2001). Updated guidelines for evaluating public health surveillance systems recommendations: recommendations from the guidelines working group.

[14] Lowrance D, Filler S, Makombe S, Harries A, Aberle-Grasse J, Hochgesang M (2007). Assessment of a national monitoring and evaluation system for rapid expansion of antiretroviral treatment in Malawi. Trop Med Int Health.

[15] OMS (2010). Human resources for health observer - issue no 2.

[16] Portal do Governo de Moçambique

[17] Mate KS, Bennett B, Mphatswe W, Barker P, Rollins N (2009). Challenges for routine health system data management in a large public programme to prevent mother-to-child HIV transmission in South Africa. PLoS One.

[18] Nicol E, Dudley L, Bradshaw D (2016). Assessing the quality of routine data for the prevention of mother-to-child transmission of HIV: an analytical observational study in two health districts with high HIV prevalence in South Africa. Int J Med Inform.

[19] Gimbel S, Micek M, Lambdin B, Lara J, Karagianis M, Cuembelo F (2011). An assessment of routine primary care health information system data quality in Sofala Province, Mozambique. Popul Health Metr.

